# Philadelphia County Medical Society

**Published:** 1850-02

**Authors:** 


### PHILADELPHIA COUNTY MEDICAL SOCIETY.

*Meeting of December*, 1849.

DR. JACKSON, (late of Northumberland,) PRESIDENT, in the Chair.

### *Discussion on the Powers of Quinine in Remittent Fevers.*

Dr. Parrish remarked that, in the absence of the lecturer for the evening, he would ask the attention of the Society to a subject of much practical interest, in the hope of eliciting inquiry, and of promoting discussion.

It is well known that, within a few years past, the practice of administering quinine in the early stage of malarial fevers of the remittent type, has become quite prevalent in those sections of the United States where these fevers are most prevalent. The idea is now extensively entertained, that remittent and intermittent fevers are essentially the same disease, and that the absence of distinct intermissions in the former, should not prevent the administration of the remedy which is found so effectual in controlling the latter. Hence, the physicians who hold to this opinion give quinine in large doses, and with a view to its antiperiodic qualities, in the remissions of a fever, for the purpose of preventing the exacerbations, which are known to occur at particular intervals. They do not wait until all febrile action has passed away, or until a crisis occurs in the natural course of the fever, on the fifth, seventh, or ninth day, or even later, but they commence at the outset with this potent remedy, just as they would do if no fever were present, as in the intermission of an intermittent. In both cases they would, perhaps, generally precede the quinine by a brisk mercurial cathartic, and then attack it with quinine, in the interval of the paroxysms, with a view to cutting short the disease.

The results of this practice, as reported to us by many highly respectable physicians of the west and south, whose experience has been ample, is, that remittent fevers which, under the plan of treatment formerly in vogue, were accustomed to run a protracted course, and to assume a serious aspect, are brought to a rapid termination, or converted, after two or three paroxysms, into the intermittent type, and easily cured. They say, that so far from the quinine increasing the heat, thirst, and sensorial disturbance incident to fever, that it allays these, calms the nervous system, and acts as a diaphoretic. Now, it is a point of great practical interest, to know how far these observations of our brethren in other sections correspond with experience here; and my object in introducing the subject at this time is, to ascertain this.

For myself I can state that, in common with most of those who hear me, I was educated in the doctrine, that in remittents we must be very cautious of bark or quinine, while the least febrile excitement is present.

The idea entertained here, by the most eminent medical teachers, when I was a student, was, that in remittent the febrile excitement must be reduced by the lancet and brisk purgation during the early stages, and that tonics were only to be given after the decline of the fever. Even my excellent father, who, as is well known by many here, was more favorable to the use of tonics and supporting treatment in fever than most of his contemporaries, taught in his lectures that bark and quinine were not to be given until the cleaning of the tongue, or the occurrence of critical sweats indicated the approach of convalescence; except in cases where malignant symptoms were developed, or where unusual evidences of prostration existed,—and then the article was given more for its general tonic properties, than with any prospect of cutting short the fever.

Within the past few years, however, from the large amount of testimony to the value of quinine in the early stages of remittent which has reached us through the medical journals, I have been induced to pursue this course, and have reason to be well satisfied with it. I have found that quinine given in the early stage of a remittent, after a free evacuation of the bowels by a mercurial cathartic, and when a distinct morning remission was observable, has had the effect of moderating the evening exacerbation, and, by continuing it for a few days, of bringing the fever to a rapid and favorable crisis. I have seldom given over 15 or 20 grains in the remission, and in mild cases have found even less than this to answer the purpose. Since adopting this plan I have not seen any cases of protracted remittent, running on for three and four weeks, and attended with tympanitis, subsultus tendinum, low delirium, hæmorrhage from the bowels, &c. Whether this fortunate exemption is attributable to the mode of treatment adopted, or to a diminished liability in our fevers of late years to assume this form, I cannot positively say; though I am inclined to think that the old plan of delaying the use of tonics may have contributed to the production of these prolonged cases.

If the practice recently introduced by the practitioners of the south and west, in the treatment of malarial fevers, be confirmed by experience, and established as sound—it must be admitted that a great step has been taken in practical medicine—diseases, once the dread of the inhabitants of large sections of our country, will be divested of much of their virulence, and be brought under the control of remedial measures, while our science will have achieved a grand triumph.

Dr. W. Jewell said he was an advocate for the early employment of quinine in fevers. In intermittent forms he preferred decided doses, and in remittent types he did not invariably confine his practice to grain doses. For several years he had been accustomed to administer quinine in the ordinary forms of autumnal fevers, without waiting for a complete intermission of the paroxysm. He had not witnessed any bad results therefrom. He would not venture, however, to advocate its use where there were signs of local congestions, or during a high state of inflammatory excitement; in such instances, the introduction of quinine, from its therapeutic properties, is contraindicated. An abatement of the more violent symptoms of febrile excitement would be an indication, with him, for the admission of quinine into the system. By this course he had often witnessed the cutting short of fever. He believed it to be a safe mode of practice. Of one thing he was certain, that he cured his cases of fever in a much shorter period now, than he did years ago, when he depended upon the free and frequent use of the lancet, and a complete intermission of the paroxysm, before he ventured to prescribe quinine. He seldom encountered those protracted and obstinate cases of slow convalescence, with a tedious train of nervous symptoms, and an almost insurmountable prostration of the nervous energy, often terminating in visceral obstructions, with which he had been familiar in the early years of his experience as a practitioner. Then it was that he bled day after day, during the paroxysm, until he had entirely subdued the fever; but of late years his mind and practice had undergone a change, so that his lancet lay rusting in its case, for want of frequent use in fevers.

He believed that we were too timid in the treatment of fevers by quinine; and he was glad to find that the employment of the article, without waiting for a perfect and distinct remission, was gaining friends. If the practice was not a favorite one now, he thought it would become so hereafter. As for his experience, it was decidedly in its favor; nor had he witnessed those distressing effects upon the stomach and pulse, which had been attributed to its use, during the prevalence of fever. The approach of *tinnitus aurium* and partial deafness he looked upon as an indication of its influence upon the nervous system, but by no means a dangerous result. But he would not recommend its indiscriminate use in fevers; like other therapeutic agents, it required the exercise of judgment and discretion.

In the administration of large doses of quinine in intermittent fevers he had had some experience. It was not a new practice to him. In 1825, the late Dr. J. Snowden, of this city, was accustomed to treat his intermittents with decided doses of quinine. His usual habit was, after free purging, to administer during the intermission 10 grains of the article at one time, and was very successful in the treatment of his cases. The same practice had been adopted by other practitioners. He (Dr. J.) had followed in the same path, and instead of prescribing quinine in one grain doses each hour, during the intervals of the paroxysm, he directed five grains every two hours, until 10, 15 or 20 grains had been taken, according to the peculiar temperament or condition of his patient. This practice, with him, had been very successful.

He had often continued the use of the quinine in intermittents, during the prevalence of the fever, without inconvenience to the patient, and he considered the practice a much safer one than many had been disposed to believe it. He would be happy to hear the experience of others on this subject.

Dr. Bell, in reference to the alleged novelty of giving quinine in the paroxysm of remittent fever, remarked, that it did not require a person to have attained a very advanced age to be able to witness a circle of medical doctrines, in which the old were revived as novelties. The practice described by Dr. Parrish is not, by any means, new or recent. Drs. Clark and Lind,—the first in his work on the *Diseases which prevail on Long Voyages*, &c.; and the second on the *Diseases of Warm Climates*, speak of the use of the bark during the exacerbation of the fever—not as a matter of speculation, but of good practice. These authors wrote more than three-quarters of a century ago. Others might be cited to the same purport. In Italy the practice has been long a familiar one.

Dr. Bell, when remittent and intermittent fever, with their congestive complications, prevailed around Philadelphia, twenty-seven and twenty-eight years ago, had, from his connection with the Philadelphia Dispensary, of which he was one of the physicians for more than twelve years, an opportunity of seeing much of these fevers. He gave, in some cases, various preparations of cinchona during the paroxysmal stage of remittent fevers, but without satisfactory results. When the blue mass, or calomel, was directed in alternation with the bark, better effects ensued.

Dr. B. said that, to hear some persons, one would suppose learning and experience to be incompatible with each other, and that a knowledge of the opinions of the best writers on a disease was a disqualification for treating it successfully. That some reading was of service, however, he had the means of proving to his professional brethren in his published lectures on the Practice of Physic, in which, when speaking of the treatment of congestive fever, he took some pains, and he believes not without success, to show his readers that this disease, which to most of them seemed to be new, had been in fact fully described, and its correct treatment laid down in various works, some of them, perhaps, on their own shelves. A reference to these would have taught them that, under the name of pernicious or malignant intermittents and remittents, were described their congestive fevers. Torti, among others, recommended and gave the bark in sub-continued fever of a malignant character.

As a farther illustration of the necessity of a physician knowing the history of the treatment of a disease which comes before him, Dr. B. adverted to the strange ignorance manifested by a physician of a Parisian hospital a few years ago, in his prescribing large doses of quinine for acute rheumatism. This gentleman began his experiments as if he had been the first to adopt the practice; overlooking or ignorant of the trials of Haygarth and other English physicians so many years before, on this point. A little literature might have saved the lives of some of the patients of the Parisian doctor, and the credit of the profession.

Our wonder at the administration and favorable reports of the good effects of quinine in the hot stage of periodical fevers will be abated if we look on this medicine in another light than as a tonic. It is a difficult task to classify medicines, with reference to either their physiological or therapeutical effects; and hence, while we can criticise existing arrangements, we find it difficult to offer better ones. If we must designate the operation of quinine in disease by a single term, that of *sedative* seems the most appropriate. Its power of allaying febrile irritation, in which disturbance of the nervous system is the chief element, cannot be doubted. That with this effect it should, also, when given in disproportionately large doses, exert narcotic and even toxical properties, is only what we must look for from nearly all articles of the vegetable kingdom which we are accustomed to use as sedatives. Be this as it may, Dr. B. expressed his belief that the admission of the sedative operation of quinine would go farther to harmonize the pathology of the fevers and other diseases, and its use in them, than a belief of its stimulating or tonic effects. As a mere stimulant it utterly fails us. As a tonic proper, in diseases in which there is simple asthenia and anemia, without nervous complication, it is equally unsuccessful. Indirectly, it seems to display a tonic operation by allaying and removing the nervous disorder and excitement, which kept the functions in a disturbed state, and produced indirect debility in consequence.

Our confidence in the administration of large doses of quinine in periodical fevers, and of its use in the paroxysmal period, will be increased by a knowledge of the fact, that this practice is now one of common recourse in southern Europe, in Algeria, and in the United States, by physicians who would seem to have been led to it without any prior concert or formal interchange of opinions.

Arsenic, the febrifuge effects of which are readily suggested by the mention of quinine, is another example of the difficulty of classifications of the Materia Medica. It is commonly spoken of as a tonic, and as such it is prescribed in periodical fevers; but its operation in these diseases is not explicable by regarding it in this light. Like quinine, its chief therapeutical action is on the nervous system; and like quinine, also, its action is more evidently tranquillizing than exciting; and hence its use is admissible in states of excitement of the nervous and even bloodvessel system, in which mere stimulants, and common tonics, too, containing the bitter and astringent principles, would be injurious. This remark, of course, applies to the administration of arsenic in small doses, short of any toxical effects, which last would include not only lesions of tissue and poisoning of the blood, but the milder degree of emaciation and anasarcous effusion. From the time of Fowler to the present day, the safety and good effects of arsenic in intermittent fever are familiar to English and American physicians; but it is only of late years that the same favorable opinions of this medicine are beginning to be entertained in France. For a time, under the dominion of the Broussain theory, arsenic was dreaded as a poison, the use of which was denounced under all circumstances. Dr. B. has himself heard Broussais in his lectures speak of it in these terms. Now, however, we find that, through the exertions of M. Boudin, and other medical officers of the French army, arsenic has been used to a great extent among the troops, as respects the number of the patients.

It would seem, from official returns, that not only is the safety, but also the efficacy, of the practice proved. In this last respect it is alleged to be superior even to quinine. Thus, while the mean duration of the cases of intermittent fever treated by quinine is stated to have been thirty days, that of those in which arsenic was administered was twenty-two days; and that while the relapses after the quinine were somewhat more than twelve per cent., those after arsenic were but a little more than three per cent.*

The expense of procuring quinine, and the great extent to which it is adulterated, gave increased importance to the question of a good and cheap substitute for this invaluable medicine.

The President of the Society (Dr. S. Jackson, formerly of Northumberland) was asked his opinion, as he had practised twenty-five years in a region of fevers; he gave it, and has since written it with amplification as follows:—

If any one reads this little paper, let him bear in mind that the writer intended to note so much only as would indicate his general principles of treatment; and that he confines his observations to the fever as he has known it in Northumberland or Philadelphia, and to such cases of this as consist of open and free reaction.

The remittent koinomiasmatic fever is a purely inflammatory disease in all times and places, having no tendency whatever to the typhus or typhoid, nor any possibility of being changed into either of these, in any of its stages. If there is no actual inflammation present, there is yet always a tendency to form it, provided the excitement be high or unequally directed. In all cases therefore of open free reaction, the first indication is to abstract all stimulant and tonic matter, whether medicinal or dietetic, till the fever is solved or changed into a perfect intermittent: those cases excepted which are protracted into a state of great debility, or such as are sometimes supervened by hemorrhage, congestion, or some other dangerous debilitation; and even here bark and animal food are inadmissible.

*Bleeding*, general or local or both, is nearly always necessary, or at least useful, particularly in children, in whom there is a determination to the head that will soon end in convulsions or inflammation and effusion. The state of the head in young persons will often indicate a repetition of this remedy; but of this we shall say more in our progress.

*Purges* ought to be mild, and given in divided doses. If you

*Dr. Bell, at the meeting of the Society, gave the general results of the use of arsenic in intermittent fever as above. He has since added the numeral returns of the comparative merits of this medicine and quinine to the manuscript report of his remarks.

are called in the morning, and this remedy is necessary, give oil or Epsom salts, the latter with magnesia, either of them to be assisted by enemata, so as to relieve the bowels before the exacerbation. This may be expected from one to four o'clock, and you must have it met by small doses of tartar emet. one sixteenth to one twelfth of a grain, largely diluted, every half hour. It is now very desirable that the physician should be there with his lancet; but if this be impossible, the patient must go on with his tart. emet. and hope for a bleeding on the following day. An intelligent nurse might determine whether cups might not, in the physician's absence, be applied to the nucha. Cases sometimes occur, however, in which blood may be drawn from the arm in the morning; and others are sometimes met with that absolutely require either bleeding or cups, so great is the febrile cephalalgia.

*Cold water* must be poured on the head during the exacerbation in great abundance, if there is much heat or pain therein. Let the patient hold his head over a wash-tub, and let cold water be poured thereon slowly and from a height of three or four feet, but in a very small stream long continued. If the body be generally hot, it must be sprinkled or sponged with cold water, or with warm water, to either of which a little vinegar may be added. The pouring of cold water on the head, like bleeding, is particularly important in children, on account of their cephalic predisposition. Above all things, be careful that the feet are warm.

Now, it is ten to one if the purging do not go on during the afternoon; and as the principal irritation thereof is passed during the morning, the continuance of it during the afternoon, as a mild diarrhœa, will help to weaken the fever. But should it not cease as the remission approaches, you must omit the tart. emetic; and if the patient be debilitated, and headach do not prevent, you may give some op. with ipec. and determine their action to the skin by sudorific tea. The purging ought to be stopped when there is a reasonable hope of an approaching perspiration. Op. is often a blessed medicine in this fever; but it must be generally given so as to sweat off its own intrinsic bad properties, or rather its inappropriate effects. But alas for the fever patient whose opium acts as an irritant.

Now it is needful to prepare for a loose state of the bowels the coming morning. Give then, to an adult, from 5 to 15 grs. cal.

at late bed time, preliminary to a dose of oil as early in the morning as possible, thus being careful that the irritation of purging be administered during the remission. If the bile be healthy and sufficient, or green and not offensive, you may give a small dose of oil at night instead of the calomel; but the least suspicion of hepatic derangement must be met by the mercury, and calomel is infinitely better than the blue mass.

*Drinks* should be milk warm, because they promote the kindly operation of the purgatives and tart. emetic; and if the ungovernable and silly patient will have cold water, let it be given in very small doses. Large cold drinks often bring on colic, and their transient sedation is always followed by reaction demanding another draught. Milk warm drinks are known to quench thirst better than cold, whether in sickness or health. But here the reader reclaims—"a cold drink is often followed by a free perspiration, the effervescing cold draught determines directly to the skin, and often most wonderfully composes a sick stomach: see what Dr. Wood says in his *Practice of Medicine.*" 'Tis true that a large cold drink will sometimes be followed by a temporary sweat, but not often; and if it be indulged in after the perspiration has begun, it is positively hurtful, particularly in bodies greatly debilitated. The Riverian draught is a useful medicine, if given in a state of brisk effervescence, and to patients who are not disposed to flatulence; but query—who is to give it? From three to ten are sick in the same house and there is one worn-out nurse, and most likely an ignorant and clumsy one, who has hardly recovered from her own sickness, and who is so oppressed with duties, that the "very grasshopper is to her a burden"—I could wish Dr. Wood to see this woman go round among the patients, salt of wormwood in one cup and lemon-juice in another—I think he would find her an unfortunate and clumsy chemist. As to the *neutral mixture*, which is the same thing in a vapid state, you might as well give the washings of your teacups and saucers. No—tart. emet. with warm drink is the divine remedy in this fever. You cannot give it judiciously without doing good—you cannot withhold it without doing ill. It is like the Philosophy intended by Horace which he thought would profit all sorts of people;

Æquè pauperibus prodest locupletibus æquè,

Æquè neglectum pueris senibusque nocebit.

Now by this mild treatment, assisted by many coadjuvants not necessary to name, as I am writing for the learned only and not a full treatise for them, nature will bring many deadly cases of fever to a safe and early crisis, or change them into a salutary intermittent. The most important part of the physician's office, is to watch the state of the head, for this is the seat of danger. Bleeding, with leeching the head or cups to the nucha in the very beginning of the exacerbation, must be repeated in many cases more than once, and the pulse must be kept under the sedative influence of tart. emet. Of this the dose may be gradually increased to one fourth of a grain; but be careful not to maintain a continual nausea, for this will bring both the medicine and physician into disgrace. Nor is this nausea necessary to the febrifuge operation of the tartar as may be learned from Lind, Chapman, Fordyce and others.

When at last you seem to have done all that can be done by bleeding, purging, tart. emetic, and yet the fever continues without any evidence of a coming solution, the patient becoming debilitated without a proportional subduction of fever, there is no doubt some local inflammation, most likely in the gastro-intestinal membrane, possibly in the meninges. But wherever it is, small doses of calomel must be given—not to salivate, for this would be a horrible thing, but merely to bring the system under the slightest influence possible. I have already said that calomel is to be preferred to the slow, feeble, uncertain, and often adulterated blue mass. Now as soon as the green bile begins to flow, you may hope for an amelioration of all the symptoms. But while the calomel is doing its part in the above state of things, the remedy which stands pre-eminent is the cold shower bath. If a common apparatus be not at hand, let the patient sit down in a common wash-tub and let a bucket of cold water be thrown over his head and shoulders; then let him be dried and put between blankets, taking some sudorific drinks as camphor tea or vinegar whey. In some cases this process may be preceded by a dose of op. and ipec. This bathing may be repeated every day, in the beginning of the exacerbation if the strength suffices—in the height of it, should the patient be weak; and be assured that it has happily terminated many cases of fever that threatened to run into the lowest state. Now this method of bathing may be used earlier in the fever with great advantage, but it is after evacuations have been pursued for some days that it proves pre-eminently salutary.

*Protracted Cases.*

When the physician is called late or some untoward circumstances occur when he is called early, the case may be protracted into a state of great danger. Then supervene the symptoms unfortunately called typhoid. The debility is great, the heat little, the tongue dry and red, the teeth uncovered—*dentes retecti*,* the feet cold, the head sweating. These symptoms, I say, have been *unfortunately* called typhoid. Tis true they afford the likeness of typhus, but the disease is still an inflammatory remittent and it will not bear the identical treatment of typhous fevers. In the above state of the system, bark, quinine, bitters, snakeroot, are advocated by many: I venture to discard them all, but particularly the bark and quinine—the best of remedies when properly used, the very worst when abused. Nor would I give one particle of animal food, not even chicken water, as long as the fever is continued.

Farinaceous diet and vegetable soup ought to be administered in small, frequent doses; and if stimuli are needed, wine whey, wine and brandy largely diluted, sweet sp. of nitre, carb. ammon. in julap. But when the tongue is dry and red, the sp. of turpentine is the best of all remedies and should be given in doses of 20 drops to 40 every two hours in mucilage or simply floating on cold water. Blisters to the suræ and nucha are of great service in this state and they ought to be drawn between the scapulæ, if there is any tendency to the lungs. Here moreover they act charmingly on the brain and thus do a double duty.

I need not remind the experienced practitioner how much good he may do by the use of opium, but if I had the leisure and it were necessary, I would "cudgel his brains" with respect to the turning of its operation on the skin. Combined with ipec. and given at bed time with warm camphor tea, it will procure sleep and some perspiration in the morning. A non-critical and partial sweat is not injurious in this as in typhous fevers; it affords, like issues in other diseases, an outlet to inflammatory action.

* This symptom I do not recollect seeing noted except by Persius. Sat. III—101.

This process continued nightly, you will find at last that the morning mador is indicating that nature, with your skilful aid, is about to solve the whole disease and prepare the patient for quinine. You will find him gaining a little every day, the pulse becoming slower, the countenance brighter, the tongue moist, the exacerbation daily less severe; till finally at your nocturnal visit, say midnight, you find a softness over the whole body with a fuller and slower pulse—this is the first time to think of quinine. This patient who has been *in dubio* for several days and has kept you continually uneasy, you visit the next morning at the dawn and find that he has happily turned the goal. Now give him a dose of op. or paregoric; and then two grs. of quinine every hour till he has taken from 12 to 16 grs. and the whole trouble is over—you have saved the life of a fellow creature, or rather the Science of Medicine has done it for you.

Tis vain for Dr. Forbes or Dr. Anybody to say that nature performed the cure. Nature sickened the man, nature brought him to the very brink of the grave; and there she would have left him, had she not been persuaded, importuned, or compelled, by your skill in medical science. Nature cure such cases! As well might you say that she cures a crevasse of the great Mississippi because she first made the breach and then afforded the mud wherewith to stop it. Nature acts by an organic necessity and requires to be aided by the means which a world of physicians have happily discovered. Hippocrates said the physician was the servant of nature; could he now see what physicians do, he would say that she is their servant.

Nature cures no mortal disease; that physicians cure many, we do certainly know; and we ought never to do this without feeling thankful for the means, as they certainly came down from Heaven. The cures of mortal diseases have become so common that they are not estimated, and the blessings we confer are not appreciated even by ourselves. Pious men give thanks at table that they have something to eat and say grace, the Friends hold a silent pause; in both which there is a twofold gain, "they kill two birds with one stone," they return thanks and acquire a wonderful appetite by delay. Now I should like to see this practice fairly carried out and a grace said or a pause made over every dose of bark. How different from this is their practice!—the ungrateful men make angry faces and complain that it is bitter and sticks in their teeth.

The patient has now passed the crisis and he is fortified for the present by the 14 grs. quinine against any return of the fever and against an impending intermittent, which he was poorly able to bear. He is not however free from an almost imperceptible fever arising from a little inflammation somewhere; but this will gradually wear away without bringing the system into sympathy and hence the old favorite, chicken broth, may be given and a little quinine or bark *must* be given every day in divided doses.

As to the use of quinine or bark during the continuance of fever, I must say of them as did Baglivi—*remedium damnabile et perniciosum.* Bark was given us for a specific purpose—the cure of periodicity and not for the cure of fever. Epicurus taught as we learn from Lucretius *L. IV.*—821 *et seq.* that our bodily organs as eyes and ears were not made for their present uses; but that when made, they found something to do and brought themselves into use. Were this philosopher to metempsicose into the body of some modern physician, he would probably teach, that having found bark beneficial in ague, he would therefore use it. This I believe is not orthodoxy in the present age. We must believe that Heaven gave the bark for the cure of periodicity as certainly as we believe that the eye was made to see with or the ear to hear with. It was not given to cure inflammation, obstruction, dropsy, or fever—therefore it does harm in this fever till nature assisted by the physician or the physician assisted by nature, removes these, or digests them so far that their floating morbosity seeks the pores, the kidneys, or the bowels.

That stimuli are sometimes necessary, I do not question; but they are the choice of evils, and such only ought to be used as produce a transient effect. Bark, bitters, and animal food, are most persistent in their effects, never failing to aggravate or cause inflammation. The writer cannot distinctly remember what mischief he did with bark on first setting out in life, as 38 years have since rolled by, sweeping away many reminiscenda; but within his clear recollection, he has seen enough in the practice of others, to make him confident in his opinion and stubborn as Cato—"*tenacem propositi virum.*" He does certainly remember giving it the first two or three years of his practice; but of this he has no pleasant recollection; and he must have abandoned it on account of its inutility or the mischief it did.

Let the reader bear in mind, that I am treating of remitting miasmatic fever which cannot in my opinion be ever changed into typhus; this last is a specific disease and bears a very different relation to animal food and bark—a fact which alone might prove the natural discrepancy.

But how is it, you inquire, that many eminent physicians have used bark with safety and advantage in these very fevers ? Have a little more patience and I will patiently tell you. The remittent and intermittent fevers are from one cause and ever ready to be converted into each other, but particularly the remittent into the intermittent. Now it is a fact that some remittents have, from the onset, very severe exacerbations ending in almost perfect intermissions. This is particularly the case in those whose exacerbations are worst every third day. Now in these cases there is a strong tendency to periodicity, to the forming of an intermittent, and hence the bark, if given in the sweating stage of the exacerbation, as practised by Clark and others, expends its stimulation on the skin, while its antiperiodic effects are valid in preventing or mitigating the next day's fever. In fact the remittents wherein the bark is found useful, are mere intermittents somewhat masked, hence bark is the appointed and heavenly remedy.

Thus quinine has sometimes been found to extinguish yellow fever; now this disease has been considered as allied to malignant intermittents from the peculiar remission on the third day; and in epidemics wherein this alliance is considerable, the quinine may sometimes prove fortunately useful. In malignant remittents there is known to be a distinguished tendency to intermission, and here it is therefore admissible.

In this disease, the very prostration which renders all these cases alarming, prevents that local inflammation that resists the just and salutary operation of the bark, in cases milder but more phlogistic. In fine, it is inflammation present or to come, conjoined with a fever essentially inflammatory, that prevents the proper operation of bark. In typhus fevers, this medicine may be used by an experienced hand, though they be attended by some inflammation; so it may be used in gout and rheumatism, but not with safety if an active phlogosis is on the increase.

In southern lands, there are many cases, and sometimes nearly whole epidemics, wherein there is very little inflammation and wherein too, the disease is characterized by a feeble circulation and an almost paralysed state of the whole man : here there is no fear of inflammatory action, hence the tonic powers of bark may raise the system above congestion, and its peculiar virtues may destroy the periodicity. Blessed medicine—which does more, through the ingenuity of physicians, than would seem to have been first intended. Among many proofs of this operation of bark in the South the reader may find some of the strongest in a paper by Dr. Wm. M. Boling on the fever of Alabama, *Amer. Jour. Med. Scien. New Series*, Vol. xii. 18.

But even in Southern climates bark cannot always be used with safety—witness the testimony of Mosely and Hillary, in the West Indies, and Johnson in the East, as well as many other authorities that might be adduced. Mosely, in treating of the remittent fever of the West Indies, says, *4th ed. Lond.* 1803,—" if the fever be a recent one, and has a tendency to a remittent, (he is now speaking of intermittents,) the premature use of the bark impedes the secretions, causes strictures in the capillary vessels, and fixes immovable obstructions in the brain. This I have so often seen that I wonder at writers not observing more caution in advising bark early in the remission of fevers," *p.* 192. He enumerates *in p.* 193 the evils that come from giving bark in remittent fever when there is any tendency to inflammation." He declaims, p. 190 to 193, against the giving of bark till the body is fully prepared for it by evacuations, and before the intermissions are perfect. To the improper use of bark in intermittent and remittent fevers, he imputes dementia, insanity, inflammations, and various obstructions.

Dr. James Johnson lost all his patients in the remittent fever of Bengal, till he abandoned the bark, and took to bleeding and calomel. He says, " it was with a trembling arm and a palpitating heart that I first opened a vein." See Johnson on " the endemic fever of Bengal," and above all attend to his necrotomic phænomena. By the way it must be conceded that Dr. Balfour used bark in the same fever after evacuations; but he had to confess after some years, that obstructions of the liver were very frequent, and probably existed in all cases, that hence he would recommend mercury so as to effect the mouth.

Let us hear what Hillary says of the remittent fever of Barbadoes. By bleeding, emetics, and saline drafts, the fever "was generally carried off by a critical sweat on the seventh or ninth day; or in some few it came to *intermit regularly* after that time, and then was soon cured by the bark, given with saline drafts. Though these irregular, ingeminated fevers often remitted and sometimes seemed to intermit, yet if the bark was given too soon in the disease, before it *intermitted regularly*, (as I have more than once seen,) it generally caused the fever to become continual and *mali moris*."

There are some dangerous diseases that admit of two general methods of cure—one by debilitation and the other by tonics. These will forever exercise the ingenuity of man, and give abundant occasions of vehement contention, while both parties have truth and reason on their side. But we are now treating of the fever in Pennsylvania, and therefore it would be well to consider what some Northern physicians of great reputation have said.

Pringle says, part III. ch. IV. sec. V. "the cure of the camp fever, (he means the remitting and intermitting fevers,) depends chiefly upon evacuations and a low diet: the bark is useful when there are *complete intermissions*." Descanting on venesection, he says,—"a person unacquainted with the nature of this disorder, and attending chiefly to the paroxysms and remissions, would be apt to omit this evacuation, and to give the bark prematurely, which might bring on a continued inflammatory fever. Rush's edit. p. 178.

Now mark well—this great man used bleeding, purging, and abundant doses of tartar emetic, thus preparing the system for a complete intermission. At p. 182, he says—"I come next to the bark, and shall observe, that these fevers have often such fair remissions and even with a breaking of the water, as might persuade a physician unacquainted with their nature, that they would always yield to this medicine; but he would be often disappointed. Whether it be that some inflammation hinders the bark from taking effect, or that these quotidians are not true intermittents, (as not being of a tertian or quartan form,) certain it is that they can seldom be safely stopped by it. For though the paroxysms have disappeared under its use, yet having so often seen the breast affected or a lurking fever remain after giving the febrifuge, at last I made it a rule to attempt the cure without it, or at least to delay it till, in the convalescent state, the patient required it only as a strengthener."

In page 186, when treating of a worse form of remitting fever which he calls the *marsh fever*, he says, " it was necessary after due preparation, to stop it in the first *fair intermission.*" And Dr. Rush in his note p. 182, says—" in the bilious remittents of the U. States, of the first, second, and sometimes of the third grade, the bark is ineffectual in curing them."

In p. 183, however, Pringle confesses, that when evacuations did not bring the fever to a crisis and the exacerbations were becoming worse, he gave bark, beginning "two or three hours before the sweat ended." Giving the bark during the sweating stage, if it was necessary, was a most prudent measure and accords with what we have said above. The stimulation is sweated off and the antiperiodic virtues remain. But Pringle took to the bark in these cases as a last resort, after his approved treatment had failed.

Donald Munro, Vol. II. 88 and 89, 2nd ed. says—"In the years 1761 when the fever came to remit, we were obliged for the most part to continue the diaphoretics; for although the disorder put on a remitting form, the bark had very little effect in stopping it, unless when the fever changed into a regular quotidian or tertian form." And again he says, " We tried the bark in various forms in many cases where the patient had been blooded and purged in the beginning, and had used the cooling medicines, and where the remissions were very clear, yet it had no effect in removing the disorder, except in a few cases wherein the paroxysms assumed a tertian form. For the most part, it made the patients more hot and feverish and we left it off, as it was in danger of changing the remittent into a continued fever.

Now mark what I said above—that when the bark succeeds in remittent fever, it must be when there is a strong tendency to run into the intermittent form. Now see here what Munro says page 90— " I have observed that in some seasons, it has a remarkably good effect, while in other seasons it rather seems to increase the heat and fever. In general the more distinct the remissions are, and the more the fevers of the season tend to perfect intermittents, the better effect the bark has."

The above are some of the best authorities, they were the instructors of my youth and I turn to them with pleasure—many others might be adduced, but time and space are wanting; moreover it is an invidious task to quote book after book which the reader is more likely to know than the present writer. *Vivo Romæ* said Baglivi in excuse of his opinion: the present writer may say in palliation of his ignorance, *annos viginti quinque Northumbriæ vixit*—that he lived 25 years in the *Ultima Thule* of medical science.

